# Chronotype, Life’s Essential 8, and risk of cardiovascular disease: a prospective cohort study in UK Biobank

**DOI:** 10.21203/rs.3.rs-6718332/v1

**Published:** 2025-06-03

**Authors:** Sina Kianersi, Kaitlin Potts, Heming Wang, Tamar Sofer, Raymond Noordam, Martin Rutter, Kathryn Rexrode, Susan Redline, Tianyi Huang

**Affiliations:** Brigham and Women’s Hospital and Harvard Medical School; Brigham and Women’s Hospital and Harvard Medical School; Brigham and Women’s Hospital; Harvard University; Leiden University Medical Center; University of Manchester; Brigham and Women’s Hospital and Harvard Medical School; Brigham and Women’s Hospital; National Institute on Aging

## Abstract

**Introduction:**

Individuals with an evening chronotype often experience circadian misalignment, which may disrupt health behaviors and circadian regulation of cardiometabolic functions such as blood pressure. However, the associations of chronotype with modifiable cardiovascular disease (CVD) risk factors and incident CVD are not fully understood.

**Methods:**

We conducted a prospective study in 322,777 UK Biobank participants aged 39-74 years who were free of known CVD (2006-2010). Chronotype was self-reported using a single representative question from the Morningness-Eveningness Questionnaire. The Life’s Essential 8 (LE8) score was calculated based on 8 modifiable CVD risk factors, and ranged from 0 to 100 with higher scores indicating better cardiovascular health. Incident CVD was defined as first myocardial infarction (MI) or stroke leading to hospitalization or death, identified via validated algorithms. Cox proportional hazards models estimated the association between chronotype and CVD risk, adjusted for socio-demographics, shift work, and family history of CVD. Under the causal mediation framework, we evaluated the role of LE8 in the association between chronotype and CVD risk by decomposing the total effect into natural direct effect (i.e., independent of LE8) and natural indirect effect (i.e., mediated by LE8; NIE).

**Results:**

Participants (mean age: 57) with a “definite evening” chronotype (8% of the total sample) were 79% more likely to have an overall poor LE8 score (<50 points) compared to “intermediate” type (prevalence ratio 95% CI: 1.72 - 1.85). Over a median of 13.2 years of follow-up, there were 17,584 incident CVD events (11,091 MI; 7,214 stroke). The hazard ratio (HR) for total CVD was 1.03 (0.99 - 1.07) for the “definite morning” and 1.16 (1.10 - 1.22) for “definite evening” compared with “intermediate” chronotype (P-trend: 0.10). LE8 explained 74% of the association between evening chronotype and CVD (NIE comparing “definite evening” to “intermediate: 1.11; 95% CI: 1.09, 1.13). Findings were similar when MI and stroke were examined individually.

**Conclusions:**

Compared to intermediate chronotype, evening chronotype was associated with modestly higher CVD risk, which was mainly explained by overall poorer cardiovascular health. These results suggest that individuals with evening chronotype may particularly benefit from interventions targeting CVD risk factors.

## INTRODUCTION

Cardiovascular disease (CVD) remains the leading cause of mortality globally and in the U.S. (1) In 2022, recognizing sleep duration as a critical component of cardiovascular well-being, the American Heart Association (AHA) added optimal sleep duration into their updated CVD prevention recommendation, Life’s Essential 8 (LE8) (2). Sleep, along with other cardiovascular health behaviors (e.g., diet, physical activity) and factors (e.g., blood pressure, blood lipids) are influenced by the universal circadian timing system (3). Circadian disruptions, specifically a mismatch between external cues such as light or rhythmic behaviors that drive sleep-wake cycles and the internal circadian clock (i.e., circadian misalignment), can lead to adverse cardiometabolic outcomes (3–7). However, less is known about the role of overall cardiovascular health, as measured by LE8, in the association between circadian misalignment and cardiometabolic outcomes.

Chronotype, an individual’s natural inclination for sleep-wake timing, reflects inherent circadian preferences and may be a marker for underlying circadian misalignments (8). Approximately 8–11% of middle-aged to older adults exhibit an evening chronotype, characterized by late-night bed time and peak activity later in the day, and about 24–35% have a morning chronotype, with earlier bedtimes and peak activity in the morning; the rest fall somewhere in between (6, 9). Compared to those with an intermediate chronotype, adults with an evening chronotype (and plausibly those with a strong morning chronotype) are at higher risk of circadian misalignment due to the mismatch between their internal circadian rhythms and external natural and social environment, such as work schedules (10). Circadian misalignment can disrupt behavioral rhythms and impair reward-related brain functions (11), potentially promoting unhealthy lifestyle behaviors such as irregular sleep patterns (12), poor diet quality, heavy alcohol drinking (13), and smoking (14). Furthermore, by altering expression of clock genes, disrupting hypothalamic–pituitary–adrenal (HPA) axis activity, impairing metabolic regulation, and increasing sympathetic nervous system activation, circadian misalignment might increase the likelihood of adverse blood pressure, glucose, and lipid profiles (3, 5, 7). Lastly, recent Genome-Wide Association Studies have identified 351 SNPs for self-reported chronotype, including variants associated with higher risks of cardiometabolic outcomes (9). These findings suggest a shared genetic architecture between chronotype and CVD. Therefore, genetic predisposition to CVD may modify the observed association between self-reported chronotype and CVD risk.

Epidemiologic and animal studies suggest that an evening chronotype is associated with a higher risk of CVD (3, 15, 16). However, the mechanisms underlying this association remain unclear. A recent umbrella review of 14 systematic reviews on observational studies and 11 Mendelian randomization (MR) studies investigating the association between sleep traits, cardiometabolic risk, and CVD concluded that the role of chronotype in these associations is inconclusive (17). Notably, no study has examined the impact of chronotype on the comprehensive measure of cardiovascular health as assessed by LE8, nor evaluated the potential mediating role of LE8 in the chronotype-CVD association. Given the circadian system’s critical role in regulating rest-activity cycles, eating habits, sleep patterns, and physiological processes (some of which are components of LE8), chronotype may affect cardiovascular outcomes through its influence on LE8.

**Figure 1:**
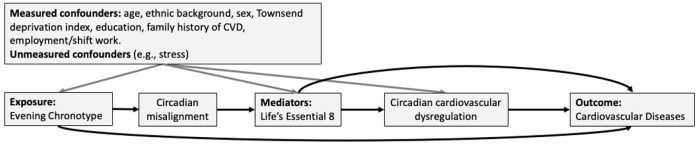
Conceptual framework on the association between chronotype and cardiovascular diseases The objectives of the study were to 1) evaluate the association between chronotype and cardiovascular health (Life’s Essential 8), 2) assess the extent to which cardiovascular health mediates the relationship between chronotype and cardiovascular diseases.

In this study, we hypothesized that individuals with an evening chronotype would be more likely to have an unfavorable cardiovascular health profile characterized by lower LE8 scores, which would increase their CVD risk (Fig. 1). Specifically, we aimed to 1) evaluate the associations of chronotype with the LE8 score and its individual components, and 2) examine the extent to which LE8 mediates the prospective association between chronotype and CVD risk.

## METHODS

### Study Design, Setting & Participants

The UK Biobank (UKB) began in 2006 with around 502,650 UK participants aged 40–69 at enrollment (18). As of the July 2023 data release (data release version 16.1), it includes 502,128 participants, after excluding withdrawals up to February 2025. Participants were recruited through the UK National Health Service, which provides coverage for 98% of the population. Approximately 9 million individuals from Great Britain (England, Scotland, and Wales) were invited by mail to participate in the UKB, with around 5.5% accepting the invitation (18). Between 2006 and 2010, participants attended one of the 22 assessment centers across Great Britain, where they consented to the study, completed a touch-screen questionnaire, participated in interviews, had their blood pressure measured, underwent physical measurements (including standing height and weight), and provided blood and urine samples. DNA extracted from blood samples was genotyped using the UK BiLEVE array for 10% of participants and the UKB Axiom array for the remaining, targeting approximately 800,000 genetic markers; through imputation, this process derived 96 million genetic variants, with quality control measures ensuring their reliability and accuracy (19). Participants are followed for disease occurrence through linkage to various UK healthcare data sources.

We implemented a prospective cohort study design using assessment center visit as our study baseline. We followed STROBE and AGReMA reporting guidelines to report our findings (20, 21). Among the 502,128 participants in the UKB dataset, 6,172 were excluded due to missing chronotype (exposure) information, 18,292 were excluded due to a history of myocardial infarction (MI) or stroke at baseline, and 154,887 were excluded because of incomplete data on one or more lifestyle behaviors or factors (primarily physical activity; Supplemental Fig. 1). Participants included in the study exhibited modestly better cardiovascular health compared to those excluded (Supplemental Table 1). UKB received approval from the National Health Service North-West Multi-Centre Research Ethics Committee, and all participants gave written informed consent at the start of the study.

### Chronotype (exposure)

Chronotype was self-reported at the UKB baseline visit (2006–2010) using a single validated question from the Morningness-Eveningness Questionnaire (MEQ) (22, 23): “Do you consider yourself to be? Definitely a ‘morning’ person; More a ‘morning’ than ‘evening’ person; More an ‘evening’ than a ‘morning’ person; Definitely an ‘evening’ person; Do not know; Prefer not to answer.” The single question’s correlation coefficient with the full MEQ score surpasses 0.72 (24, 25), and its stability ranges from moderate [kappa = 0.626 over 6 years in Nurses’ Health Study II (6)] to substantial (kappa > 0.72 over an average of 10 years in the UKB; Supplemental Fig. 2). This simple assessment has been adopted in other large cohort studies, such as the Nurses’ Health Studies (6), for its empirical validation for assessing chronotype in middle-aged to older populations. Responses of ‘Prefer not to answer’ were treated as missing data. However, in the main analyses, consistent with a previous study (9), we classified ‘Do not know’ responses as intermediate chronotype (n = 28,688). To address potential misclassification and align with prior studies (6, 26), we also grouped “More a ‘morning’ than ‘evening’ person” and “More an ‘evening’ than a ‘morning’ person” into this intermediate category. Thus, the chronotype variable had three categories: definitely morning, intermediate, and definitely evening.

### Life’s Essential 8 (mediators)

LE8 framework comprises four health behaviors—diet, physical activity, nicotine exposure, and sleep health—and four health factors: body mass index (BMI), blood lipids, blood glucose, and blood pressure, totaling eight components. Each LE8 component has a scoring algorithm that assigns a score between 0 and 100, with higher scores indicating better cardiovascular health in that component. These individual scores are then averaged to generate an unweighted composite LE8 score, ranging from 0 to 100, where a higher score represents better overall cardiovascular health. In alignment with AHA’s scoring criteria, we created scores for each LE8 component as well as a composite LE8 score (2).

Diet was calculated based on dietary information gathered at baseline using the UKB touchscreen questionnaire. Consistent with previous studies, a healthy diet was defined by higher intakes of fruits, vegetables, and fish, and lower consumption of processed and red meats (27, 28). The total minutes spent on walking, moderate, and vigorous physical activities were self-reported using questions adapted from the International Physical Activity Questionnaire (29, 30). Typical sleep duration was self-reported using a single touchscreen question, and we coded outlier values (< 2 hours and > 14 hours) as missing. Nicotine exposure was assessed based on self-reported smoking status, years since quitting, and whether the participant lived with an active indoor smoker.

Certified nurses and healthcare technicians measured standing height with a Seca 202 stadiometer (Hamburg, Germany) and weight using a Tanita BC418MA body composition analyzer (Tokyo, Japan) or a standard scale; these measurements were used to calculate BMI (kg/m^2^) (31). Blood samples were collected and analyzed for total cholesterol, high-density lipoprotein (HDL) cholesterol, triglycerides, low-density lipoprotein (LDL) direct, and glycated hemoglobin (HbA1c) using standardized protocols. Total cholesterol was measured by CHO-POD analysis, HDL cholesterol by enzyme immuno-inhibition, LDL direct by enzymatic protective selection, and triglycerides by GPO-POD analysis; all on a Beckman Coulter AU5800 analyzer (Beckman Coulter (UK) Ltd.) (32, 33). Similar to previous studies (34), non-HDL cholesterol was calculated by subtracting HDL cholesterol from total cholesterol values. For missing HDL cholesterol values, we calculated non-HDL cholesterol using LDL direct and triglycerides, applying a modified Friedewald equation, [non-HDL cholesterol = LDL direct + (Triglycerides ÷ 2.2)], with all values expressed in mmol/L (35). HbA1c levels were assessed using the Bio-Rad Variant II Turbo analyzer, utilizing high-performance liquid chromatography. Rigorous quality control procedures were implemented to ensure consistency and precision across all assays (32, 36).

Resting systolic and diastolic blood pressures were measured twice, with a one-minute interval between readings, using an Omron 705 IT monitor (Omron Healthcare Europe B.V., Hoofddorp, the Netherlands) (37), and we calculated the mean for analysis. When automated measurements were unavailable, we incorporated values from manual readings. Medications for hypertension, dyslipidemia, and type 2 diabetes were recorded through nurse-led interviews. Lastly, prevalent diabetes cases, needed for calculating blood glucose score, were identified based on participants’ health records, medication use, HbA1C level, and self-reports, following prior studies (38, 39). Supplemental Table 2 lists the UKB data fields we used to derive each LE8 component and the overall score.

### Cardiovascular Disease (CVD; outcomes)

Incident CVD was defined as a composite of fatal and nonfatal MI and/or any stroke events. We chose to focus on MI and stroke primarily because they are “hard” CVD outcomes with more definitive clinical diagnoses. In contrast, softer CVD events (e.g., angina, heart failure without hospitalization) often have more diagnostic variability, which can introduce misclassification bias. The UK Biobank Outcome Adjudication Group developed and validated algorithms using UKB self-reported medical conditions, International Classification of Diseases (ICD-9 and ICD-10) codes linked to hospital admission data, and ICD-10-coded death registry records to ascertain a range of health outcomes among UKB participants, including MI and stroke (40). A systematic review found that the positive predictive values (PPVs) for identifying MI or its subtypes through electronic health records generally ranged from 70–100% when validated against reference standards, such as chart reviews (40, 41). Systematic reviews by the UK Biobank Stroke Outcomes Group showed that the PPVs for identifying stroke or its major pathological types from hospital and death registry data were at least 66%, validated against reference standard data sources (e.g., reviews by a specialist physician), though the accuracy of self-reported data was lower and inconsistent (40, 42, 43). The majority of MI and stroke cases were identified from linked health records.

### Covariates

Age, ethnic background, sex, education, family history of CVD along with employment/shift work status were self-reported at study baseline (2006–2010) using the touchscreen questionnaire. ‘Do not know’ and ‘Prefer not to answer’ responses were set to missing values. Employment/shift work status was based on whether participants reported paid employment and, if so, how often they worked outside a typical 9am–5pm schedule; those not in paid employment, including retirees, were grouped as a separate category. The Townsend Deprivation Index was calculated using data from the most recent national census output areas available prior to each participant’s enrollment in the UKB. Genetic risk was considered as a potential effect modifier. A previously conducted study generated a polygenic risk score (PRS) for CVD and made it available in the UKB, with increased PRS scores indicating a higher CVD risk (44, 45). The PRS was developed using genome-wide summary statistics derived from a meta-analysis of independent genome-wide association studies, applying a Bayesian based approach. We categorized participants into low, intermediate, and high-genetic risk groups for CVD based on PRS tertiles.

### Statistical Analysis

We employed Poisson regression with robust error variance to calculate prevalence ratios (PRs) for the associations between chronotype and unfavorable cardiovascular health profiles measured by LE8 component and overall scores (46). Following prior research and AHA guidelines, scores ≥ 50 points were classified as ‘favorable,’ and < 50 as ‘unfavorable’ (2, 6). Model 1 was adjusted for age, ethnic background, sex, Townsend deprivation index, education, and family history of CVD. Model 2 was further adjusted for employment/shift work. In additional models, to evaluate the association of each LE8 component independently, we adjusted for the other seven LE8 components.

We used Cox proportional hazards models to estimate hazard ratios (HRs) for the association between chronotype and CVD risk, with person-time at risk accruing from baseline (2006–2010) until date of CVD diagnosis, loss to follow-up, death, or the censoring date of May 31, 2022. In these prospective analyses, we fitted two models as described above, and additionally adjusted model 2 for the overall LE8 score or each of its eight components. We found no evidence of a violation of the proportional hazards assumption, as indicated by the non-significant interaction term between follow-up time and chronotype for incident CVD outcome (P for interaction in Model 2: 0.25). We further conducted these analyses separately for MI and stroke.

We used a causal framework to evaluate the mediating role of LE8 on chronotype-CVD association. This approach allows for decomposition of the total effect of chronotype on CVD into the natural direct effect (independent of LE8) and the natural indirect effect (mediated by LE8) in the presence of an exposure-mediator interaction (47, 48). We used a causal mediation SAS macro to estimate the proportion of the association explained by the overall LE8 score (47). As associations with incident CVD were observed only for the “definitely evening” chronotype, compared to “intermediate,” the mediation analysis was limited to this comparison (N = 244,554). Mediation analyses assumed no unmeasured confounding for chronotype-CVD, LE8-CVD, or chronotype-LE8 associations, and that LE8-CVD confounders were unaffected by chronotype. The LE8-CVD (mediator-outcome) causal association has been previously established (2). To assess potential bias from unmeasured confounding, we calculated E-values for chronotype-LE8 (exposure-mediator) and chronotype-CVD (exposure-outcome) associations (49, 50). Of note, although LE8 components may have changed over time, previous studies in UKB indicate that these changes are generally modest to high. For instance, about 44% of the 31,344 UKB participants reported consistent physical activity levels across two time points (51), while BMI demonstrated greater stability, with measurements four years apart showing a strong correlation (*r* = 0.93) (39, 52).

We conducted a series of sensitivity analyses to test the robustness of our findings: 1) To understand the role of sex on the cross-sectional associations, we performed subgroup analyses by sex; 2) To assess the impact of coding the “Don’t Know” response for chronotype as intermediate, we excluded this response category and recalculated the HRs for the chronotype–CVD associations; 3) Given that CVD was likely underdiagnosed during the COVID-19 pandemic (53), we reset the censoring date to March 23, 2020 (the start of the national lockdown in the UK) and repeated the prospective analyses; 4) We repeated the prospective and mediation analyses separately for MI and stroke subtypes to explore potential differences in associations; 5) To evaluate potential effect modification by age groups, sex, employment/shift work status, dichotomized overall LE8 score, and CVD PRS tertiles on the chronotype–CVD associations, we conducted subgroup analyses and evaluated interactions on the multiplicative scale; and 6) To better understand potential selection bias introduced by excluding participants with missing data on LE8, we analyzed the chronotype–CVD associations in the entire UKB sample, adjusting for covariates in Models 1 and 2. We used Python (version 3.12.4) for data processing and visualization and SAS version 9.4 (Cary, NC) for data analyses.

## RESULTS

The mean (SD) baseline age of the study population was 57 (8) years. Around 96% of participants identified as White, 47% were male, 37% held a college or university degree, and 58% had a family history of CVD (Table 1). Around 9% reported working in roles involving shift work. The mean (SD) of LE8 score was 67 (12), with 7.2% classified as having unfavorable cardiovascular health (LE8 overall score <50). Most participants (67%) had an intermediate chronotype, while 8% reported a “definitely evening” chronotype. Compared to “intermediate” chronotypes, those with a “definitely evening” chronotype were younger, had lower socioeconomic status (indicated by a higher Townsend Deprivation Index), were more likely to hold a college or university degree (43% vs. 37%), more frequently engaged in shift work (12% vs. 9%), and had a lower overall LE8 score (65 vs. 68). In descriptive analyses, for seven of the eight LE8 components, participants with a “definitely evening” chronotype also had notably poorer scores compared to those with an “intermediate” chronotype. Conversely, compared to the ‘intermediate’ group, those with a ‘definitely morning’ chronotype had marginally poorer scores on five components. The distribution of chronotype was similar between sexes; however, women had a higher (i.e., healthier) overall LE8 score than men (70 vs. 65 points), with particularly higher scores in diet (61 vs. 46) and blood pressure (48 vs. 36; Supplemental Table 3).

### Associations between chronotype and LE8

In cross-sectional analyses, compared to the “intermediate” chronotype, participants with a “definitely evening” chronotype were 79% (PR 95% CI: 1.72 - 1.85) more likely to have a poor cardiovascular health profile (LE8 score <50) after adjusting for covariates in model 2 (Table 2). In contrast, “definitely morning” chronotype participants were 5% less likely to have a poor overall LE8 score (PR: 0.95, 95% CI: 0.92 - 0.98). In fully adjusted models (model 2 + remaining LE8 components), “definitely evening” chronotype was associated with a higher likelihood of poor scores in six of the eight LE8 components compared to the “intermediate” group except for blood pressure and blood lipid components. The strongest associations were seen for nicotine exposure (PR: 1.54, 95% CI: 1.50 - 1.58) and inadequate sleep (PR: 1.42, 95% CI: 1.36 - 1.48). Associations for “definitely morning” chronotype and individual LE8 components were less consistent, with higher likelihood of a poor sleep score (PR: 1.30, 95% CI: 1.26 - 1.34) but lower likelihood of a poor diet score (PR: 0.88, 95% CI: 0.87 - 0.89) compared to the “intermediate” chronotype. The associations between chronotype and poor blood pressure or blood lipid scores were null or negligible. Associations between chronotype and overall LE8 score were stronger among female compared to male participants (PR comparing “definitely evening” to “intermediate” chronotype: female: 1.97, 95% CI: 1.86 - 2.09; male: 1.67, 95% CI: 1.60 - 1.75); p-interaction: 0.0003; Supplemental Table 4).

### Associations between chronotype and incidence CVD

A total of 17,584 incident CVD cases (MI = 11,091; stroke = 7,214) were documented over a median follow-up of 13.2 years (IQR = 1.5). Compared to participants with an “intermediate” chronotype, those with a “definitely evening” chronotype had a 16% higher risk of CVD (HR: 1.16, 95% CI: 1.10 - 1.22), while participants reporting “definitely morning” chronotypes were not at higher risk (HR: 1.03, 95% CI: 0.99 - 1.07) after adjustment for age, ethnic background, sex, Townsend deprivation index, education, family history of CVD, and employment/shift work status (Table 3). Associations attenuated after further adjusting for the eight LE8 components. Similar findings were observed for MI and stroke individually (Table 3), or when alternative chronotype groupings were used, excluding “Don’t Know” responses (Supplemental Table 5). Setting the follow-up endpoint to March 2020 slightly strengthened associations (Supplemental Table 6). Associations were also stronger for specific stroke subtypes (ischemic stroke and subarachnoid hemorrhage), though some subtypes had smaller case numbers, making these estimates less stable (Supplemental Tables 7-8). In subgroup analyses, no significant interactions were found between chronotype and age, sex, employment/shift work, LE8 score, or CVD PRS for their association with incident CVD (p-interaction > 0.11; Supplemental Table 11). However, the association appeared stronger among older participants, men, non-shift workers, or those with an LE8 score ≥50. Similar associations were observed across CVD PRS groups.

### Mediation role of LE8 on “definitely evening” chronotype and CVD association

**Figure 2:**
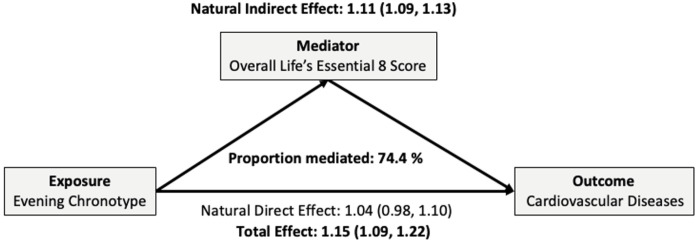
Mediation role of overall Life’s Essential 8 score on the chronotype and incident cardiovascular diseases association, N = 244,554 Adjusted for age, ethnic background, sex, Townsend deprivation index, education, family history of CVD along with employment/shift work. In mediation analyses, we used a reverse-coded version of LE8 scores with 0 showing healthiest lifestyle. As associations with incident CVD were observed only for the “definitely evening” chronotype, compared to “intermediate,” the mediation analysis was limited to this comparison (N = 244,554).

Based on model 2, the HR (95% CI) for the total effect of chronotype on incident CVD, comparing “definitely evening” to “intermediate”, was 1.15 (1.09, 1.22). The slight discrepancy between the total effect estimates from the Cox proportional hazards model (HR = 1.16) and the mediation macro (HR = 1.15) likely reflects differences in how ties (exact vs. Breslow method) and confounders were handled in each model. The natural indirect effect, representing the effect mediated by the overall LE8 score, was 1.11 (1.09, 1.13), indicating that 74% of the association between chronotype and incident CVD was mediated through the LE8 score. No direct effect of evening chronotype on CVD was observed, with an HR of 1.04 (0.98, 1.10) (Figure 2). In separate mediation analyses evaluating each LE8 component individually, nicotine exposure emerged as the strongest mediator, accounting for 34% of the association between evening chronotype and CVD, followed by sleep (14%), blood glucose (12%), body weight (11%), and diet (11%) (Supplemental Table 9). Similar patterns were observed for MI and stroke, with the mediation effect of the overall LE8 score being especially pronounced for incident MI (Supplemental Table 10).

The E-value (lower 95% CI) for the association between chronotype and the LE8 overall score was 2.98 (2.83), suggesting that an unmeasured confounder would need to be associated with both by a PR of at least 2.98 (2.83) to fully explain the observed association, conditional on covariates in model 2. This high E-value makes such confounding unlikely. However, the E-value for the HR of the chronotype–CVD association was lower (1.59; lower 95% CI = 1.43), indicating that unmeasured confounding bias may not be ruled out.

## DISCUSSION

Among middle-aged to older adults, evening chronotype was cross-sectionally associated with an overall poor LE8 score (< 50 points). This association was particularly strong among women and was observed for six of the eight LE8 components. Conversely, a morning chronotype, compared to an intermediate chronotype, was associated with a slightly lower likelihood of having an overall poor LE8 score, particularly in the components of diet, physical activity, and nicotine exposure. In prospective analyses including a median of 13 years of follow-up, evening chronotype was associated with a modestly increased risk of CVD compared to intermediate chronotype. However, most of this association was explained by a poor overall LE8 score, with nicotine exposure being the strongest individual mediator. No natural direct effect of chronotype on CVD was observed. Lastly, chronotype–CVD associations were not modified by sociodemographic factors or genetic susceptibility to CVD.

Similar to our findings, a smaller study of 506 women reported an association between evening chronotype and a poor AHA Life’s Simple 7 score, the earlier version of LE8 that excluded sleep and used simpler metrics for other components, such as not accounting for secondhand smoking (54). Most existing research has either focused on individual cardiovascular risk factors (55), or considered chronotype as one component of broader sleep health (56, 57). Systematic reviews and meta-analyses have identified associations between evening chronotype and individual metabolic CVD risk factors, including elevated blood glucose and blood lipids (55, 58). Consistent with our findings, no association was observed with blood pressure (55). However, contrary to our findings, a meta-analysis of 33 studies found no association between chronotype and BMI, although heterogeneity was high across studies (55). Nonetheless, in a large cohort of over 63,000 female nurses, we previously observed an association between evening chronotype and higher BMI, as well as current smoking, suboptimal sleep duration (< 7 or ≥ 9 h/d), lower physical activity levels, and low diet quality (6).

Consistent with our finding of a modest association between evening chronotype and higher CVD risk (MI and/or stroke), prior prospective studies have reported similar associations for different CVD outcomes (e.g., coronary heart disease or more broadly defined CVD [ICD-10: I00–I99]) in the UK Biobank or other cohorts (15, 58). These studies often assessed chronotype as part of a composite sleep score or analyzed a simplified dichotomous chronotype variable (evening versus morning chronotype) (16, 56, 57, 59, 60). Given that chronotype exists on a continuum from morningness to eveningness, with intermediate in between (10), the dichotomous approach may introduce misclassification bias by overlooking the intermediate group, where the majority of the population fall. The three-level categorical chronotype (morning, evening, intermediate) more accurately depicts the chronotype construct and may better capture differences in underlying circadian misalignment.

We further identified three MR studies examining these associations (59, 61, 62). A one-sample MR analysis in the UKB suggested a slightly increased risk of acute MI for morning chronotype (compared to evening), with weak statistical support (61). In contrast, two two-sample MR studies based on summary statistics found no association between morning chronotype and MI (62) or stroke (59). However, all these MR studies used genetic markers derived from a UKB GWAS designed to identify markers associated with the morning versus non-morning chronotype (i.e., combining the evening and intermediate chronotypes as the reference) (9). As a result, these studies were unable to identify genetic associations for evening chronotype, and the findings were not directly comparable to observational studies that compare the morning or evening chronotypes with the intermediate chronotype. Importantly, genetic markers for the evening chronotype may not fully overlap with those for the non-morning chronotype.

We found that LE8 mediated a large majority of the association between chronotype and CVD risk, with no direct effect of chronotype on CVD outcomes. There are several potential mechanisms that may explain the observed mediation between chronotype and cardiovascular health-related factors. As highlighted earlier, individuals with an evening chronotype often experience circadian misalignment and show behavioral rhythms that may be misaligned with day and night light patterns—a central zeitgeber for control of circadian rhythms (3, 10). Individuals with extreme chronotypes may experience irregularity of various behaviors relevant to cardiovascular health such as irregular eating schedules, lower levels of physical activity, and inadequate or irregular sleep, some of which further exacerbate the misalignment (3). Misaligned light exposure and behaviors also may disrupt the central as well as the peripheral molecular circadian clocks present in cells across vascular, cardiac, and endocrine systems (3). Clock genes (e.g., BMAL1, CLOCK, PER, CRY) influence metabolic pathways in peripheral tissues; disrupted expression of these genes can trigger inflammation, endothelial dysfunction, and disruption in heart rate (3, 7). Further, circadian misalignment can affect the hypothalamic-pituitary-adrenal axis and the peripheral clocks in key metabolic organs, such as the liver and pancreas, thereby increasing sympathetic activity and elevating blood pressure (3, 5). These changes alter glucose metabolism, reduce insulin sensitivity, decrease leptin, disrupt daily cortisol rhythm, foster weight gain, and overall exacerbate cardiometabolic risk (4, 5). Additionally, delayed melatonin secretion due to extended late-night light exposure in evening chronotypes further disrupts the alignment of these metabolic hormones (4, 5). Taken together, individuals with an evening chronotype are more prone to circadian misalignment, increasing their susceptibility to behavioral and biological changes that impair cardiovascular health and in long-term elevate CVD risk (2, 3, 10).

### Strengths

Our study is the first to evaluate the mediating role of LE8 in the chronotype–CVD association. The prospective design, with minimal loss to follow-up (n = 752), ensured the temporal sequence of exposure and outcome, strengthening causal inference compared to cross-sectional designs. The simultaneous assessment of multiple health behaviors and factors via the LE8 framework provided a holistic examination of modifiable CVD risk factors. Objective evaluations of LE8 factors (blood measurements and weight), along with validated exposure and outcome assessments, reduced potential measurement errors. Furthermore, the large sample size reduced random error, enhanced statistical power, and allowed for adjustment for a comprehensive set of confounders. Where possible, we used continuous measures to mitigate residual confounding. Lastly, we conducted multiple sensitivity analyses to evaluate the robustness of the main findings.

### Limitations

There are several study limitations as well as factors that may mitigate these. Reliance on a single chronotype question could cause misclassification despite the high correlation with the overall MEQ score (24, 25). Chronotype was assessed only at a single time point. However, chronotype is partly influenced by genetics (9) and showed high stability in our study (kappa of 0.72) when measured more than 10 years apart. The LE8 variables were measured at the same time as chronotype (exposure), limiting our ability to ensure that potential mediators strictly follow the exposure in a temporal sequence. Nonetheless, previous studies suggest that lifestyle variables remain relatively stable over time within the UK Biobank cohort (39, 51, 52). Self-reported data may lead to measurement error, but validation studies have extensively confirmed the reliability of these measures, as outlined in our methods. Given the low response rate during UKB recruitment (~ 5.5%), selection bias is a potential concern (18). However, the direction and magnitude of the associations observed in nationally representative samples were similar to those derived from the UKB cohort (63). Because UKB includes predominantly White, healthier participants and may not be a random sample of the general population, generalizability of our findings is limited to this segment of the population. While included participants had a slightly healthier overall LE8 score, the magnitude and direction of associations between chronotype and CVD, after adjusting for key covariates (model 2), remained consistent in both the full UKB sample (N = 477,664) and our study cohort (Supplemental Table 12). Lastly, our mediation analysis of the overall LE8 score as the primary mediator did not fully account for potential multi-step mediation, where one LE8 component may influence another. To address this, we conducted eight separate mediation analyses for each LE8 component.

## Conclusion

Our study among middle-age to older UK adults suggests strong associations between an evening chronotype and a poor overall LE8 score, particularly among women. Moreover, our prospective findings suggest that the association between evening chronotype and CVD risk is primarily mediated by a poor overall LE8 score. We further found evidence that LE8 components each individually mediate the evening chronotype–CVD association. These findings highlight evening chronotype as a clinically relevant factor for informing CVD mitigation strategies, and specifically, for targeting promotion of AHA LE8 among people with an evening chronotype. Additionally, our findings provide a foundation for future MR and mechanistic studies to explore the pathways underlying the relationship between chronotype and CVD risk.

## Figures and Tables

**Table 1.** Characteristics of participants at baseline by chronotype in the UK Biobank (2006-2010)

		Chronotype		
	Total sample N = 322,777	Definitely morning N = 78,223	Intermediate N = 217,857	Definitely evening N = 26,697
**Age (years), Mean (SD)**	56.5 (8.1)	57.5 (7.9)	56.4 (8.1)	55 (8.3)
**White ethnic background, %**	95.8	94.9	96.2	94.9
**Male, %**	47.0	44.7	47.7	48.0
**Townsend deprivation index, Mean (SD)**	−1.5 (3)	−1.4 (3)	−1.6 (2.9)	−1 (3.2)
**College or University degree, %**	36.6	33.6	36.9	43.3
**Family history of stroke or heart disease, %**	57.9	58.6	57.8	57.0
**Employment/shift work, %**				
Employed, never/rarely shift work	49.5	47.6	50.1	49.9
Employed, sometimes/usually/always shift work	9.3	8.9	9.1	11.6
Retired/Other	41.3	43.4	40.8	38.5
Life’s Essential 8 components *				
**Diet score, Mean (SD)**	53.7 (31.6)	57.4 (31.2)	52.9 (31.5)	48.9 (31.8)
**Physical activity score, Mean (SD)**	72.1 (27.8)	74.5 (27.1)	71.9 (27.7)	66.8 (29.6)
**Nicotine exposure score, Mean (SD)**	72.2 (30.2)	74.3 (29)	72.4 (29.9)	64.5 (35.1)
**Sleep health score, Mean (SD)**	89.9 (18.1)	88.4 (19.2)	90.7 (17.3)	87.4 (19.9)
**Body weight score, Mean (SD)**	70.3 (28)	69.4 (28.4)	71 (27.7)	67.2 (29.4)
**Blood lipids score, Mean (SD)**	47.7 (29.1)	47.6 (28.9)	47.8 (29.1)	47.4 (29.4)
**Blood glucose score, Mean (SD)**	90.6 (19.5)	89.7 (20.1)	91 (19.2)	89.7 (20.6)
**Blood pressure score, Mean (SD)**	42.5 (32.2)	41.1 (31.9)	42.8 (32.3)	44.1 (32.4)
**Life’s Essential 8 overall score, Mean (SD)**	67.4 (11.7)	67.8 (11.7)	67.6 (11.6)	64.5 (12.5)

* Life’s Essential 8 components were scored as points, with higher values reflecting closer adherence to recommended cardiovascular health behaviors and factors.

**Table 2.** Cross-sectional associations between chronotype and Life’s Essential 8 components, N = 322,777

	Unhealthy lifestyle / Sample size	PR (95% CI) Model 1*	PR (95% CI) Model 2†	PR (95% CI) Model 2 + Mutual adjustment‡
Diet score <50				
Definite morning	22,098 / 78,223	**0.87 (0.86 - 0.88)**	**0.87 (0.86 - 0.88)**	**0.88 (0.87 - 0.89)**
Intermediate	73,125 / 217,857	Ref.	Ref.	Ref.
Definite evening	10,359 / 26,697	**1.14 (1.12 - 1.15)**	**1.13 (1.12 - 1.15)**	**1.08 (1.06 - 1.10)**
P-trend	105,582 / 322,777	**<.0001**	**<.0001**	**<.0001**
Physical activity score <50				
Definite morning	17,328 / 78,223	**0.88 (0.87 - 0.89)**	**0.88 (0.86 - 0.89)**	**0.88 (0.87 - 0.90)**
Intermediate	54,768 / 217,857	Ref.	Ref.	Ref.
Definite evening	8,508 / 26,697	**1.25 (1.23 - 1.27)**	**1.26 (1.24 - 1.29)**	**1.19 (1.17 - 1.21)**
P-trend	80,605 / 322,777	**<.0001**	**<.0001**	**<.0001**
Nicotine exposure score <50				
Definite morning	7,913 / 78,223	**0.89 (0.87 - 0.91)**	**0.89 (0.87 - 0.92)**	**0.92 (0.90 - 0.94)**
Intermediate	24,783 / 217,857	Ref.	Ref.	Ref.
Definite evening	5,342 / 26,697	**1.63 (1.59 - 1.67)**	**1.62 (1.58 - 1.66)**	**1.54 (1.50 - 1.58)**
P-trend	38,038 / 322,777	**<.0001**	**<.0001**	**<.0001**
Sleep score <50				
Definite morning	5,849 / 78,223	**1.28 (1.24 - 1.32)**	**1.29 (1.25 - 1.33)**	**1.30 (1.26 - 1.34)**
Intermediate	12,005 / 217,857	Ref.	Ref.	Ref.
Definite evening	2,279 / 26,697	**1.53 (1.47 - 1.60)**	**1.50 (1.44 - 1.57)**	**1.42 (1.36 - 1.48)**
P-trend	20,133 / 322,777	0.1119	**0.0155**	**<.0001**
Weight score <50				
Definite morning	18,601 / 78,223	**1.09 (1.07 - 1.11)**	**1.09 (1.07 - 1.11)**	**1.09 (1.07 - 1.10)**
Intermediate	46,688 / 217,857	Ref.	Ref.	Ref.
Definite evening	7,095 / 26,697	**1.24 (1.21 - 1.27)**	**1.24 (1.21 - 1.26)**	**1.14 (1.12 - 1.17)**
P-trend	72,384 / 322,777	**0.0201**	**0.0318**	0.0641
Blood lipids score <50				
Definite morning	44,350 / 78,223	**0.99 (0.98 - 0.998)**	**0.99 (0.98 - 0.997)**	**0.99 (0.98 - 0.998)**
Intermediate	123,315 / 217,857	Ref.	Ref.	Ref.
Definite evening	15,219 / 26,697	**1.03 (1.02 - 1.04)**	**1.03 (1.02 - 1.04)**	1.01 (0.999 - 1.02)
P-trend	182,885 / 322,777	**<.0001**	**<.0001**	**0.0007**
Blood glucose score <50				
Definite morning	3,936 / 78,223	**1.06 (1.03 - 1.10)**	**1.07 (1.03 - 1.11)**	1.04 (0.999 - 1.07)
Intermediate	9,516 / 217,857	Ref.	Ref.	Ref.
Definite evening	1,545 / 26,697	**1.36 (1.29 - 1.44)**	**1.35 (1.28 - 1.42)**	**1.17 (1.11 - 1.23)**
P-trend	14,997 / 322,777	**<.0001**	**0.0001**	**0.0249**
Blood pressure score <50				
Definite morning	40,801 / 78,223	**1.02 (1.01 - 1.02)**	**1.02 (1.01 - 1.02)**	**1.00 (1.00 - 1.01)**
Intermediate	108,304 / 217,857	Ref.	Ref.	Ref.
Definite evening	12,706 / 26,697	**1.01 (1.00 - 1.03)**	1.01 (0.999 - 1.02)	**0.99 (0.97 - 0.999)**
P-trend	161,811 / 322,777	**0.0168**	**0.0104**	**0.0160**
Life’s Essential 8 overall score <50				
Definite morning	5,184 / 78,223	**0.95 (0.92 - 0.98)**	**0.95 (0.92 - 0.98)**	Not applicable
Intermediate	14,756 / 217,857	Ref.	Ref.	Not applicable
Definite evening	3,284 / 26,697	**1.80 (1.74 - 1.86)**	**1.79 (1.72 - 1.85)**	Not applicable
P-trend	23,224 / 322,777	**<.0001**	**<.0001**	Not applicable

* Model 1: adjusted for age, ethnic background, sex, Townsend deprivation index, education, family history of CVD

† Model 2: adjusted for covariates in model 1 along with employment/shift work.

‡ Adjusted for Model 2 + the other seven LE8 components.

*P*-trend was calculated by modeling chronotype as a continuous variable ranging from 1 to 3. All outcomes were defined as having a component score of less than 50 in the corresponding Life’s Essential 8 criteria.

**Table 3.** Chronotype and cardiovascular diseases risk in UK Biobank, N = 322,777

	Chronotype HR (95 CI)			*p*-trend
	Definitely ‘morning’	Intermediate	Definitely ‘evening’	
**Cardiovascular disease (MI and/or stroke)**				
Cases / person-years	4,525 / 993,796	11,576 / 2,780,188	1,483 / 338,560	17,584 / 4,112,545
Model 1 *	1.03 (0.995, 1.07)	Ref.	**1.16 (1.10, 1.23)**	0.06
Model 2 †	1.03 (0.997, 1.07)	Ref.	**1.16 (1.10, 1.22)**	0.10
Model 2 + overall Life’s Essential 8 score	**1.05 (1.01, 1.08)**	Ref.	1.05 (0.99, 1.11)	0.25
Model 2 + all Life’s Essential 8 components	1.03 (0.99, 1.07)	Ref.	**1.06 (1.00, 1.12)**	0.86
**Myocardial infarction (MI)**				
Cases / person-years	2,847 / 1,000,347	7,304 / 2,797,135	940 / 340,669	11,091 / 4,138,151
Model 1 *	1.04 (0.99, 1.08)	Ref.	**1.16 (1.08, 1.24)**	0.22
Model 2 †	1.04 (0.995, 1.09)	Ref.	**1.15 (1.08, 1.23)**	0.31
Model 2 + Life’s Essential 8 score	**1.05 (1.01, 1.10)**	Ref.	1.03 (0.96, 1.10)	0.12
Model 2 + all Life’s Essential 8 components	1.03 (0.99, 1.08)	Ref.	1.04 (0.97, 1.12)	0.64
**Stroke**				
Cases / person-years	1,861 / 1,006,106	4,739 / 2,811,862	614 / 342,682	7,214 / 4,160,651
Model 1 *	1.02 (0.96, 1.07)	Ref.	**1.19 (1.09, 1.29)**	**0.045**
Model 2 †	1.02 (0.96, 1.07)	Ref.	**1.18 (1.09, 1.29)**	0.06
Model 2 + Life’s Essential 8 score	1.03 (0.98, 1.09)	Ref.	**1.10 (1.01, 1.20)**	0.65
Model 2 + all Life’s Essential 8 components	1.02 (0.97, 1.08)	Ref.	**1.10 (1.01, 1.20)**	0.45

* Model 1: adjusted for age, ethnic background, sex, Townsend deprivation index, education, family history of CVD

† Model 2: adjusted for covariates in model 1 along with employment/shift work.

The Life’s Essential 8 score and its individual components are continuous measures developed in accordance with the American Heart Association guidelines. They range from 0 to 100, where a higher point indicates better cardiovascular health. *P*-trend was calculated by modeling chronotype as a continuous variable ranging from 1 to 3.

Values in rows corresponding to models represent hazard ratios (95% CI) for the association between chronotype and outcome.

Follow-up time was included as person-days in the Cox proportional hazards models. For the table, this was converted to person-years by dividing by 365.25.
